# Columnar apple primary roots share some features of the columnar-specific gene expression profile of aerial plant parts as evidenced by RNA-Seq analysis

**DOI:** 10.1186/s12870-014-0356-6

**Published:** 2015-02-04

**Authors:** Romina Petersen, Haris Djozgic, Benjamin Rieger, Steffen Rapp, Erwin Robert Schmidt

**Affiliations:** Department of Molecular Genetics, Johannes Gutenberg-University, Mainz, D-55128 Germany

**Keywords:** Primary root, Homozygous, Columnar, Apple, Gypsy-44, RNA-Seq, DESeq, MapMan, qRT-PCR

## Abstract

**Background:**

Primary roots (radicles) represent the first visible developmental stages of the plant and are crucial for nutrient supply and the integration of environmental signals. Few studies have analyzed primary roots at a molecular level, and were mostly limited to *Arabidopsis*. Here we study the primary root transcriptomes of standard type, heterozygous columnar and homozygous columnar apple (*Malus* x *domestica*) by RNA-Seq and quantitative real-time PCR. The columnar growth habit is characterized by a stunted main axis and the development of short fruit spurs instead of long lateral branches. This compact growth possesses economic potential because it allows high density planting and mechanical harvesting of the trees. Its molecular basis has been identified as a nested Gypsy-44 retrotransposon insertion; however the link between the insertion and the phenotype as well as the timing of the phenotype emergence are as yet unclear. We extend the transcriptomic studies of columnar tissues to the radicles, which are the earliest developmental stage and investigate whether homozygous columnar seedlings are viable.

**Results:**

Radicles mainly express genes associated with primary metabolism, growth and development. About 200 genes show differential regulation in a comparison of heterozygous columnar radicles with non-columnar radicles, whereas the comparison of homozygous columnar radicles with non-columnar radicles yields about 300 differentially regulated genes. Genes involved in cellulose and phenylpropanoid biosynthesis, cell wall modification, transcription and translation, ethylene and jasmonate biosynthesis are upregulated in columnar radicles. Genes in the vicinity of the columnar-specific Gypsy-44 insertion experience an especially strong differential regulation: the direct downstream neighbor, *dmr6-like*, is downregulated in heterozygous columnar radicles, but strongly upregulated in columnar shoot apical meristems.

**Conclusions:**

The transcriptomic profile of primary roots reflects their pivotal role in growth and development. Homozygous columnar embryos are viable and form normal radicles under natural conditions, and selection towards heterozygous plants most likely occurs due to breeders’ preferences. Cell wall and phytohormone biosynthesis and metabolism experience differential regulation in columnar radicles. Presumably the first step of the differential regulation most likely happens within the region of the retrotransposon insertion and its tissue-specificity suggests involvement of one (or several) tissue-specific regulator(s).

**Electronic supplementary material:**

The online version of this article (doi:10.1186/s12870-014-0356-6) contains supplementary material, which is available to authorized users.

## Background

Columnar apple trees show a characteristic pillar-like growth habit with a thick, stunted main axis and short lateral fruit spurs [1,2]. This growth habit could be of potential benefit to apple growers because columnar trees can be planted closer together and require less pruning than standard tree types [2,3]. However, none of the columnar cultivars available to date can compete with commercially successful cultivars in terms of fruit quality and disease resistance [2-7]. Columnar growth arose as a spontaneous somaclonal mutation of a McIntosh tree in Canada in 1961 [8,9]. With one exception [10], all columnar cultivars that have been described so far are heterozygous (hemizygous) for the columnar mutation (*Co*/-) [11]. Whether the lack of homozygous individuals (*Co*/*Co*) is due to a decreased viability of homozygous columnar seeds/seedlings or just the decision of apple growers to preferentially choose non-columnar breeding partners is unclear.

The molecular cause of the columnar phenotype has recently been identified as the insertion of a Gypsy-44 long terminal repeat (LTR) retrotransposon into the LTR of another retrotransposon on chromosome 10 [10]. Wolters et al. [12] detected a smaller columnar-specific insertion at the same position, which is the solo-LTR of Gypsy-44 and thus most likely represents an artefact (unpublished data). The Gypsy-44 insertion is probably responsible for the upregulation of a nearby gene encoding a 2OG-Fe(II) oxygenase (also called *downy mildew resistance 6-like* (*dmr6-like*)) of unknown function in apical meristems and axillary buds of columnar trees, whereas leaves do not show any expression of *dmr6-like* [10,12]. Overexpression of *dmr6-like* in *Arabidopsis thaliana* led to a columnar-like phenotype [12]. In addition to the upregulation of *dmr6-like*, a significant increase of the expression level of other genes within the retrotransposon vicinity has been shown [10,13]. The consequence is a change in the overall gene expression pattern of the columnar plants. The shoot apical meristems and leaves of columnar apple trees show a differential regulation of defense-associated genes, genes involved in secondary metabolism such as terpene and phenylpropanoid synthesis, as well as genes related to auxin and jasmonate synthesis and signaling [10,13,14]. Since a reliable detection of the columnar growth habit is only possible after about two to three years, it is as yet unclear at which developmental time point the gene expression patterns leading to the formation of the columnar habit are established. Up to now, it is also not known whether and how the gene expression pattern of roots is affected by the *Co* mutation. Even the phenotype of own roots of columnar apple trees has never been analyzed, which is probably due to the fact that the vast majority of columnar trees are grown as scions on non-columnar rootstocks.

Germination and radicle emergence are the first developmental steps towards the formation of a new plant. The radicle is important for anchorage, nutrient and water supply of the plantlet as well as for the perception and integration of a multitude of environmental signals such as gravity or pest attacks. Its tip has even been described as the “brain” of the plant by Charles Darwin [15] (cited in [16]). Despite their crucial regulatory and pioneering role in development, little research has been conducted on primary roots at the molecular level (e.g. deciphering their transcriptome profiles) and those were mainly limited to the model plants *Arabidopsis thaliana* and *Zea mays* (for example [17-21]). Only one publication has dealt explicitly with the transcriptome of adult roots of poplar [22]. While apple seeds have been the subject of several studies mostly owing to their deep and well-pronounced dormancy [23-28], research interest seems to fade significantly when they finish germination. Apple seed dormancy can be overcome by cold treatment (stratification) for 60 – 90 days depending on the cultivar and environmental conditions [23,28]. After this time, the radicle protrudes the testa as the primary root. Primary roots of the dicotyledonous model plant *Arabidopsis* consist of four longitudinal sections: the root cap (columella) at the tip, followed by the zone of division, the elongation zone and the differentiation zone [29]. A cross section of the root reveals a radial organization of different cell layers: epidermis, cortex, endodermis and the vascular cylinder encompassing the pericycle, protoxylem, protophloem and procambium [30,31]. This radial symmetry is established at the root apical meristem, a small set of cells near the root tip surrounding the mitotically less active quiescent center [32].

In this study we analyzed and compared the transcriptomes of heterozygous columnar, homozygous columnar and non-columnar primary apple roots. Our aims were 1) to gather general information about the gene expression profile of this poorly studied plant tissue, 2) to analyze whether homozygous columnar seedlings exist and are viable, 3) to determine whether the columnar-specific gene expression profile observed in aerial plant parts can already be observed at the earliest stages of development in the root and 4) to further investigate how the Gypsy-44 insertion might be linked to the formation of the growth phenotype. For this purpose, apple seeds were subjected to stratification and radicles were harvested when they had reached a length of about 3 cm. Half of the radicle was used for DNA isolation and subsequent genotyping, while the remaining half containing the root tip was used for RNA isolation followed by Illumina sequencing. The transcriptomic reads were assembled and contigs were subjected to Basic Local Alignment Search Tool (BLAST) searches [33] as well as Blast2GO analyses [34,35] to gain a comprehensive view of the genes expressed in primary apple roots in general. Furthermore, the reads were mapped to the apple draft genome [36] and individual gene expression levels (normalized read counts) were compared between columnar and non-columnar radicles, with a special focus on the genes in the vicinity of Gypsy-44, the *Co* mutation. Gene expression patterns in the *Co* target region were compared across primary roots, leaves and shoot apical meristems by additional quantitative real-time PCRs (qRT-PCRs). Similarities and differences in the gene expression patterns of the different tissues were found. Our data make a substantial contribution to the understanding of the development of the columnar growth habit and primary root function in general.

## Results

### Homozygous columnar apple seedlings are viable

**Figure 1:**
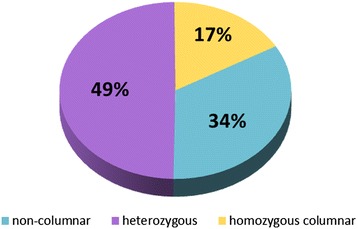
**Genotypes of primary roots.** The genotype of 119 apple primary roots was determined by marker PCRs with regard to the presence of the Gypsy-44 retrotransposon insertion.

To investigate whether homozygous columnar apple seedlings show reduced viability or phenotypic effects compared to standard-type seedlings in early developmental stages, seeds obtained from apples of the heterozygous columnar cultivar ‘Procats 28’ (P28) that had been subjected to open pollination were germinated. As the trees were grown surrounded by other columnar apple varieties, the chance of pollination by a columnar father was high. After about 12 weeks of incubation at 4°C, the germination rate of the seeds was approximately 80%. No obvious phenotypic differences could be observed between individual radicles. The radicle genotype with regard to the presence of the columnar-specific Gypsy-44 transposable element (TE), which most likely represents the original *Co* mutation [10], was determined via PCRs. The diagnostic PCR assays as established by Otto et al. discriminate unambiguously between the non-columnar, the heterozygous columnar and the homozygous columnar genotype [10]. Of 119 seedlings subjected to genotyping, 40 seedlings were detected to be non-columnar, 59 showed a heterozygous columnar genotype, while the remaining 20 seedlings carried the columnar-specific Gypsy-44 insertion homozygously. These results could be confirmed by PCR assays using our indel-based markers I2_3_M1 and H1_M1 that are tightly linked to the *Co* mutation [37]. In total, a genotype ratio of non-columnar : heterozygous columnar : homozygous columnar seedlings of 2 : 3 : 1 (Figure 1) was detected. This suggests that homozygous columnar apple embryos are viable and most likely germinate at normal ratios.

### Primary roots mainly express genes for growth, development and signaling

We sequenced RNA extracted from one primary root of each genotype and obtained about 118 million, 104 million and 126 million reads for the non-columnar, the heterozygous columnar and the homozygous columnar sample, respectively [EMBL: PRJEB6212] (Table 1). In order to investigate biological replicates, in a second approach, three radicles of each genotype were pooled prior to RNA isolation and were subjected to Illumina sequencing, yielding about 40 million, 28 million and 67 million reads for the non-columnar, the heterozygous columnar and the homozygous columnar sample, respectively [EMBL: PRJEB6212]. Illumina reads were assembled in the CLC Assembly Cell using different k-mer sizes, and assemblies yielding the highest N50 value were used for downstream analysis. For the first datasets comprising more than 100 million sequences, the highest N50 values were obtained for k = 17, for the smaller replicate datasets for k = 18. Table 1 summarizes the assembly results. In the larger datasets, about 44,000 – 49,000 contigs were produced, whereas assemblies of the smaller datasets yielded about 28,000 – 38,000 contigs. N50 values were in the range of 1,200 – 1,500 bp. With no mismatches allowed, more than 40% of the trimmed reads of each dataset could be mapped back to the corresponding contigs.

**Table 1:** **Results of assemblies**

	**# raw seqs**	**k-mer size**	**# contigs**	**GC content (%)**	**Longest contig**	**Shortest contig**	**N50**	**Reads mapped back (%)**
**PR (−/−)**	118400555	17	48023	43	9994	200	1484	63
**PR (Co/-)**	104464241	17	44197	44	10381	200	1455	59
**PR (Co/Co)**	125731385	17	48932	43	16563	200	1487	60
**PRs (−/−)**	40261320	18	32471	45	11741	200	1288	43
**PRs (Co/-)**	27708416	18	28493	45	13248	200	1242	40
**PRs (Co/Co)**	67322394	18	37527	44	8970	200	1329	52

Illumina sequences were assembled and key values for contigs are indicated. The percentage of reads that was successfully mapped back to the contigs as a reference is shown in the last column.

In order to find out how many and which genes were represented in the datasets, BLAST searches were conducted with all contigs against the annotated apple genes (MDPs), *Malus* x *domestica* expressed sequence tags (ESTs), *Malus* x *domestica* unigenes and the SwissProt/UniProtKB database (Table 2 and Table 3). A maximum of 89% of the sequences matched to a homolog in the MDP list, whereas only up to 60% of the contigs had a match against the SwissProt/UniProtKB database. Only up to 19 contigs had the same hit in the MDP database (meaning they most likely represent fragments of the same gene), indicating that about 89% of the contigs represent different genes. By contrast, up to 266 contigs yielded the same SwissProt/UniProtKB hit, thereby reducing the number of individual genes detected to about 11,500 (44% of the contigs created).

**Table 2:** **Results of BLASTx searches against MDPs and Malus ESTs**

	**MDPs**		**Malus ESTs**	
	**# contigs with hits (% of total contigs)**	**# different hits (% of contigs with hits)**	**# contigs with hits (% of total contigs)**	**# different hits (% of contigs with hits)**
**PR (−/−)**	40010 (83)	25197 (63)	38094 (79)	31484 (83)
**PR (Co/-)**	37666 (85)	23966 (64)	35816 (81)	30183 (84)
**PR (Co/Co)**	40110 (82)	25100 (63)	38208 (78)	31583 (83)
**PRs (−/−)**	28377 (87)	19456 (69)	26746 (82)	23621 (88)
**PRs (Co/-)**	25278 (89)	17481 (69)	23550 (83)	21262 (90)
**PRs (Co/Co)**	32067 (85)	21350 (67)	30071 (80)	26012 (87)

The number and percentage of contigs yielding a hit and the number of different hits are indicated. The percentage of different hits refers to the total number of contigs and thus represents an estimate for the percentage of contigs representing individual genes.

**Table 3:** **Results of BLASTx searches against Malus Unigene and SwissPro/UniProtKB**

	**Malus Unigene**		**SwissProt/UniProt KB**	
	**# contigs with hits (% of total contigs)**	**# different hits (% of contigs with hits)**	**# contigs with hits (% of total contigs)**	**# different hits (% of contigs with hits)**
**PR (−/−)**	31593 (66)	17379 (55)	26221 (55)	11467 (44)
**PR (Co/-)**	29806 (67)	16883 (57)	24992 (57)	11238 (45)
**PR (Co/Co)**	31636 (65)	17288 (55)	26044 (53)	11404 (44)
**PRs (−/−)**	22545 (69)	14437 (64)	19102 (59)	9821 (51)
**PRs (Co/-)**	19923 (70)	13398 (67)	17111 (60)	9253 (54)
**PRs (Co/Co)**	25278 (67)	15373 (61)	21343 (57)	10331 (48)

The number and percentage of contigs yielding a hit and the number of different hits are indicated. The percentage of different hits refers to the total number of contigs and thus represents an estimate for the percentage of contigs representing individual genes.

Replicate datasets were subjected to Blast2GO analysis. Of the sequences yielding BLAST hits against SwissProt/UniProtKB, 51% – 63% were successfully annotated. About 86,000 – 100,000 Level 2 gene ontology (GO) terms were assigned (see Additional file 1), half of which were associated with growth and development (categories “metabolic process”, “growth”, “developmental process” and “cellular component organization or biogenesis”) or with the integration and reaction to environmental signals (categories “response to stimulus”, “biological regulation” and “signaling”). This is in line with the major roles of primary roots.

### Differential gene expression in columnar versus non-columnar radicles is widespread

For evaluation of differential gene expression we chose a mapping approach rather than an assembly approach. For each sample, reads were mapped against the annotated Golden Delicious genome [36], total read counts for each MDP were extracted, and differential gene expression across the three genotypes was statistically analyzed in DESeq [38]. Highly expressed (base mean > 100), significantly up- or downregulated genes (fold change > 2 or < 0.5 and p-value < 0.05) were visualized in MapMan [39]. A Venn diagram showed high overlap of active genes (represented as MDPs to which at least one read was mapped) between the three genotypes (see Additional file 2). Furthermore, all libraries showed a high degree of correlation, with Pearson correlation coefficients ranging from 0.80 – 0.98, indicating that overall gene expression was similar across genotypes and that replicate datasets showed consistency for gene expression values (Table 4).

**Table 4:** **Pearson correlation coefficients of RNA-Seq libraries**

	**PR (−/−)**	**PRs (−/−)**	**PR (Co/-)**	**PRs (Co/-)**	**PR (Co/Co)**	**PRs (Co/Co)**
**PR (−/−)**	1.00	0.84	0.80	0.83	0.85	0.86
**PRs (−/−)**		1.00	0.91	0.98	0.95	0.96
**PR (Co/-)**			1.00	0.93	0.93	0.89
**PRs (Co/-)**				1.00	0.95	0.96
**PR (Co/Co)**					1.00	0.92
**PRs (Co/Co)**						1.00

Correlation coefficients were calculated based on total read counts obtained for individual MDPs.

**Figure 2:**
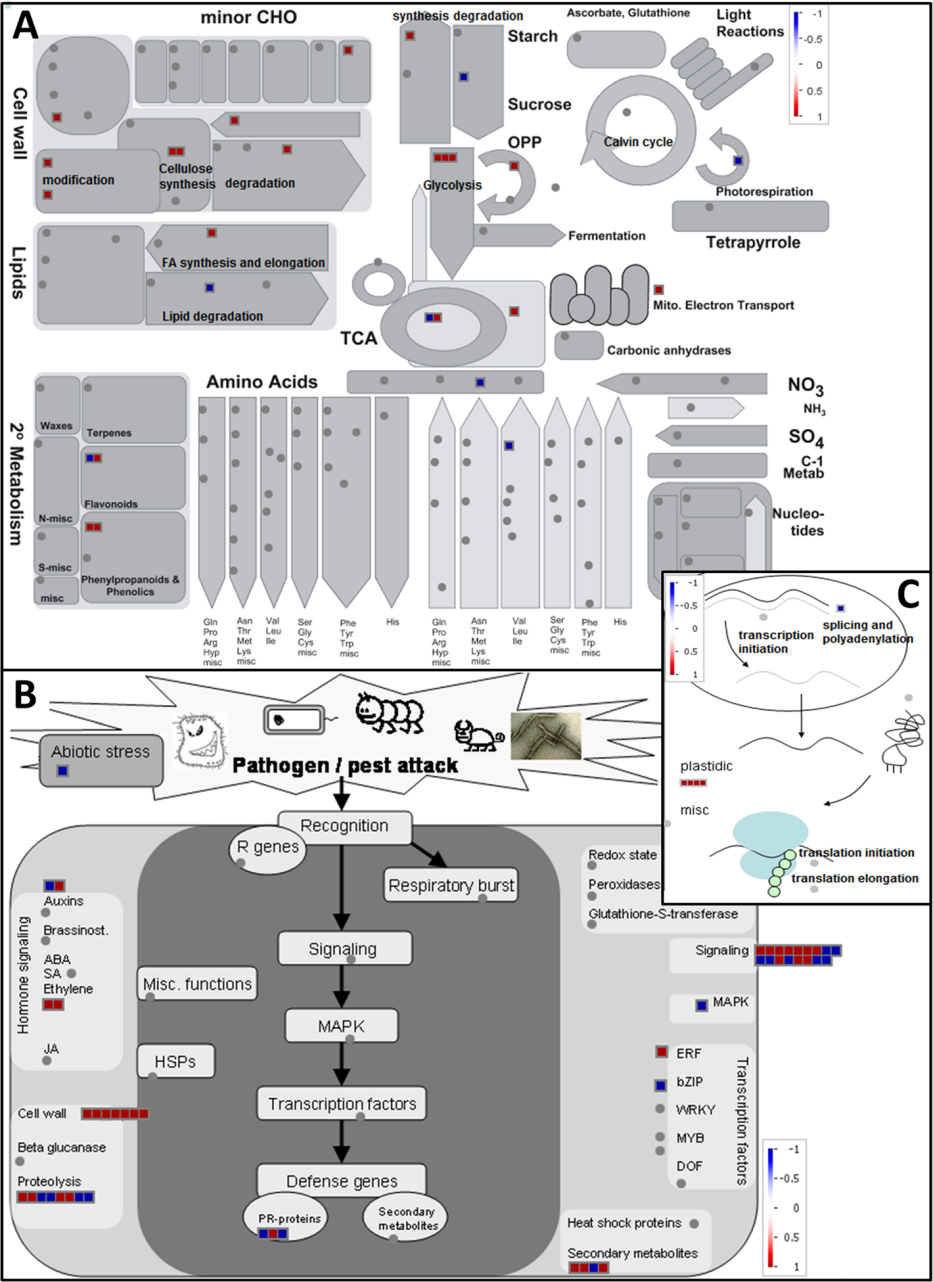
**Differential gene expression in heterozygous compared with non-columnar primary roots.** LOG2 fold changes of significantly differentially expressed genes (normalized to the non-columnar sample) as listed in Additional file 3 were imported and visualized in MapMan for the heterozygous columnar sample with regard to a metabolism overview **(A)**, pathogen/pest attack **(B)** and transcription and translation **(C)**. Genes upregulated in heterozygous columnar radicles are shown as red boxes, downregulated genes are shown as blue boxes.

The comparison of non-columnar versus heterozygous columnar primary roots identified 194 significantly differentially expressed genes (see Additional file 3). Visualization in MapMan (Figure 2) showed that many genes were upregulated in heterozygous columnar roots when compared with non-columnar roots. This applied to genes involved in cellulose synthesis, cell wall modification and degradation, glycolysis, the oxidative pentose phosphate pathway, the biosynthesis of phenylpropanoids, starch and fatty acids and the mitochondrial electron transport. On the other hand, single genes involved in lipid and starch degradation and photorespiration were downregulated in the heterozygous columnar radicles (Figure 2A). Some genes associated with stress reactions such as ethylene biosynthesis and signaling (Figure 2B) were also more highly expressed in heterozygous columnar than in non-columnar primary roots. This also holds true for genes encoding the plastidic ribosomal proteins (Figure 2C).

**Figure 3:**
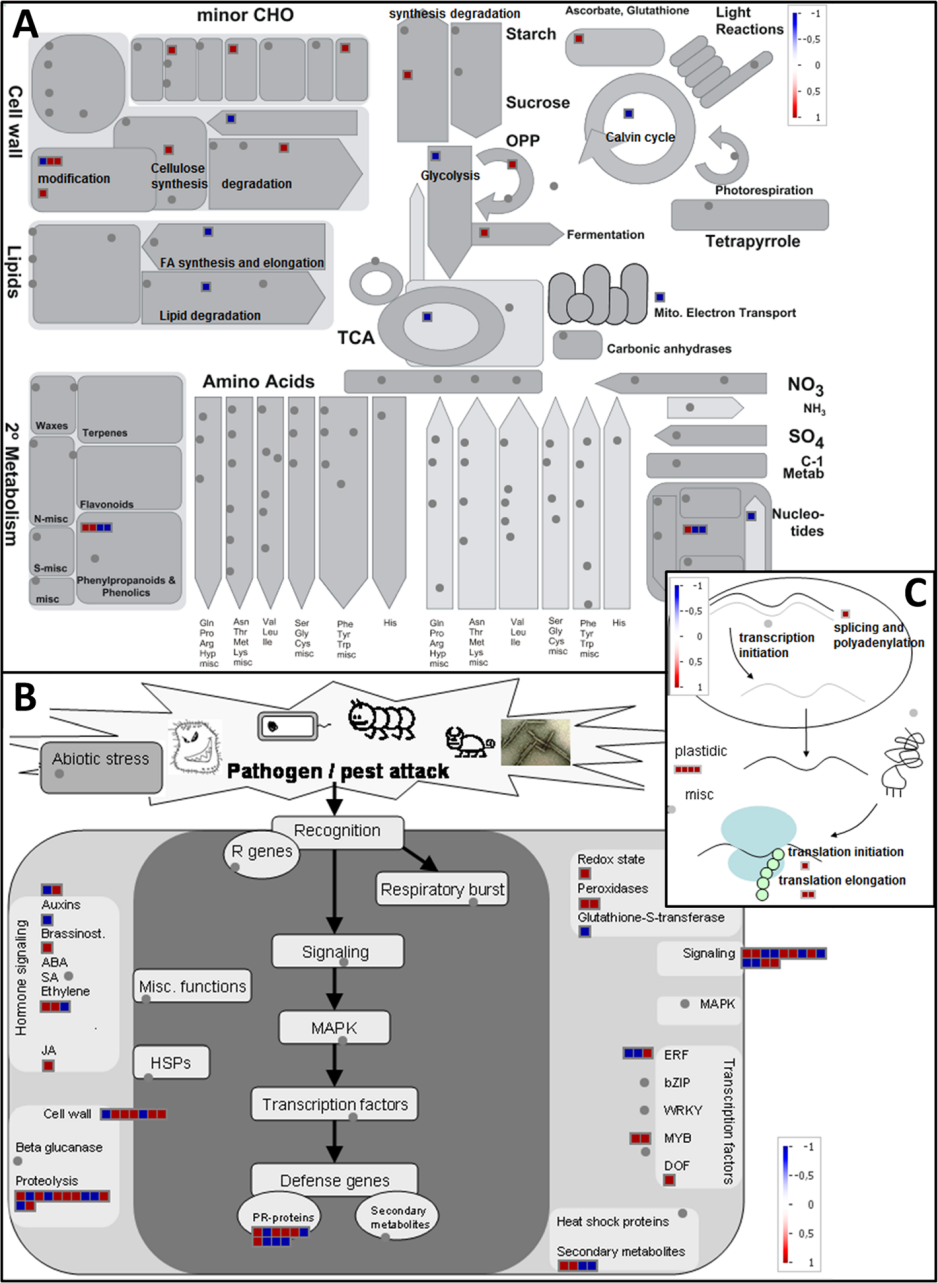
**Differential gene expression in homozygous compared with non-columnar primary roots.** LOG2 fold changes of significantly differentially expressed genes (normalized to the non-columnar sample) for the homozygous columnar sample as listed in Additional file 4 were imported and visualized in MapMan with regard to a metabolism overview **(A)**, pathogen/pest attack **(B)** and transcription and translation **(C)**. Genes upregulated in homozygous columnar radicles are shown as red boxes, downregulated genes are shown as blue boxes.

The comparison of non-columnar versus homozygous columnar primary roots yielded 269 significantly differentially expressed genes (see Additional file 4) and most of them were induced in homozygous columnar radicles (Figure 3). Single genes encoding components of the carbohydrate metabolism, cellulose synthesis, cell wall modification and degradation, the oxidative pentose phosphate pathway, fermentation, starch synthesis and ascorbate/glutathione metabolism were more highly expressed in the homozygous columnar radicles than in the non-columnar radicles (Figure 3A). On the other hand, single genes involved in fatty acid synthesis and lipid degradation, glycolysis, the tricarboxylic acid cycle, the mitochondrial electron transport chain and the Calvin cycle showed lower expression in the homozygous columnar roots than in the non-columnar primary roots. With regard to genes involved in stress reactions (Figure 3B), abscisic acid, ethylene and jasmonate-associated genes, genes encoding Myb transcription factors and peroxidases as well as genes involved in the maintenance of the redox state were upregulated. Genes linked to transcription and translation were also upregulated, and this holds true for nuclear as well as plastidic genes (Figure 3C).

When heterozygous and homozygous primary root samples were compared with each other, a similar picture emerged as for the analysis of non-columnar versus homozygous columnar radicles (see Additional file 5). 264 genes showed significant differential regulation (see Additional file 6) and of these, most genes were upregulated in the homozygous columnar primary roots. These genes are linked to primary and secondary metabolism (see Additional file 5A), stress reactions (see Additional file 5B) as well as transcription and translation (see Additional file 5C). The only exceptions were some genes involved in glycolysis and mitochondrial electron transport, which showed a lower expression in homozygous columnar radicles than in heterozygous columnar radicles.

**Figure 4:**
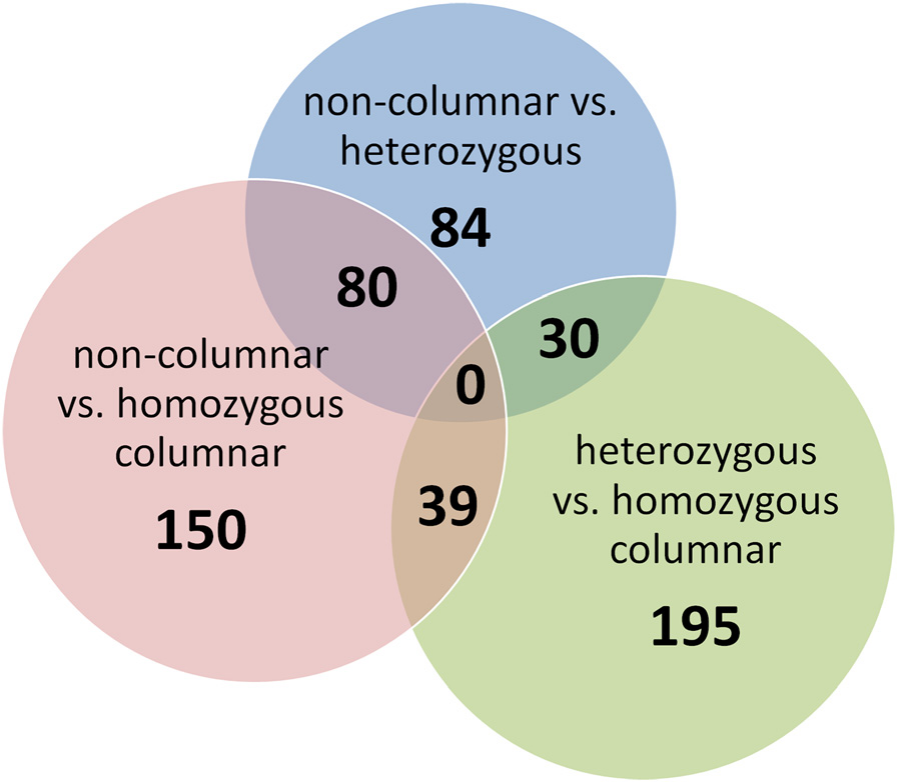
**Venn diagram summarizing the differentially expressed genes identified in the three binary comparative gene expression analyses.** While no genes were found to be differentially expressed across all three analyses, there was a high degree of overlap of the genes detected as differentially expressed in either columnar genotype versus the non-columnar genotype.

The differentially expressed genes identified in each of the three binary analyses were compared among each other (Figure 4). No genes were identified as differentially expressed in all three comparisons. 80 of the genes showing differences in gene expression between heterozygous and non-columnar radicles also show differential expression in homozygous columnar compared with non-columnar radicles. 30 of the genes identified as differentially expressed in the heterozygous columnar versus the non-columnar radicles were also differentially expressed in the heterozygous versus the homozygous columnar radicles. Another 39 genes differentially expressed in the heterozygous versus the homozygous columnar radicles were also differentially expressed in the homozygous columnar versus non-columnar radicles.

### Genes downstream of Gypsy-44 are downregulated in radicles but upregulated in aerial organs of columnar plants

**Figure 5:**
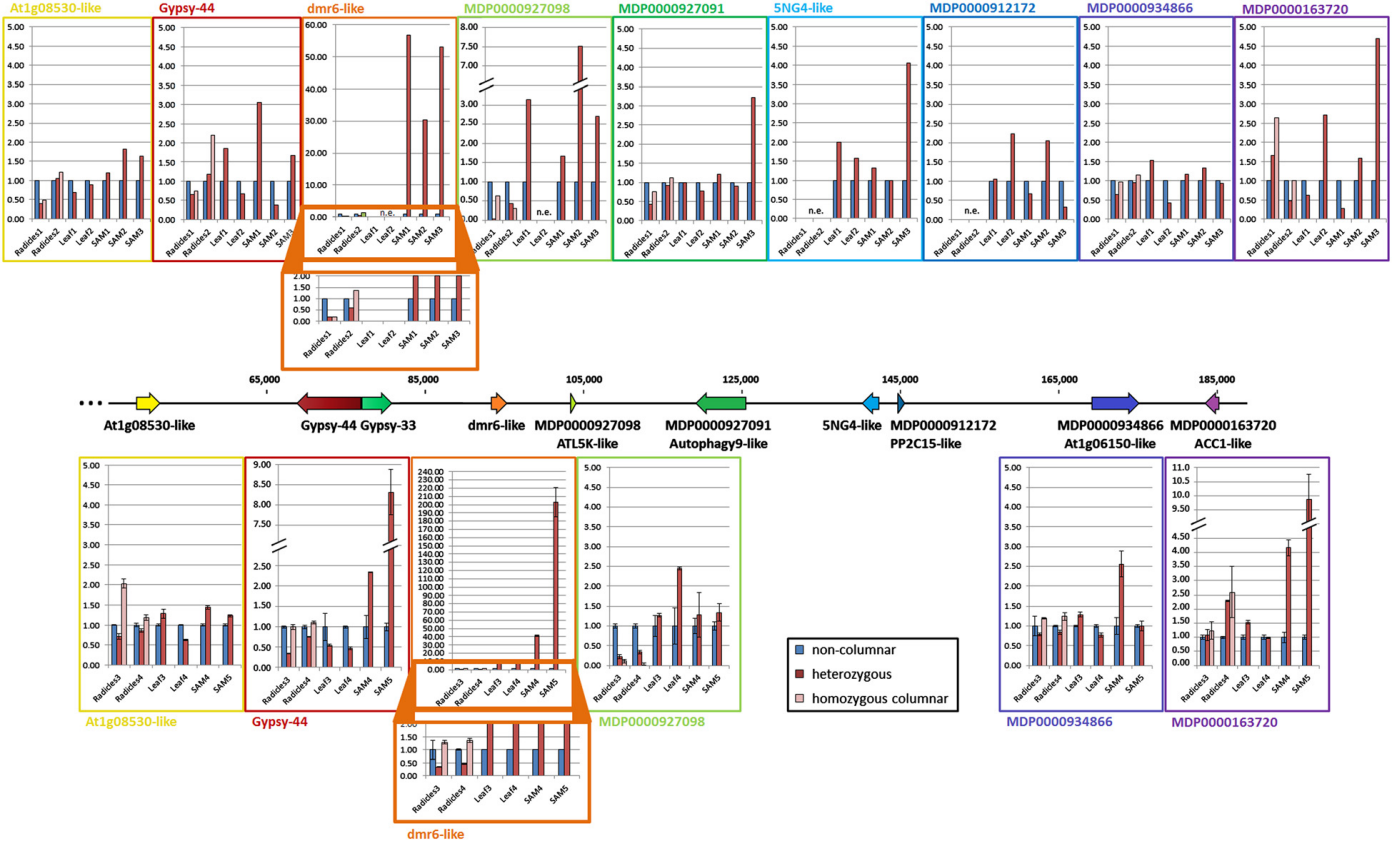
**Differential gene expression in the** ***Co*** **target region.** Fold changes of RNA-Seq samples (upper panel) and n-fold expression of qRT-PCR experiments (lower panel) in different tissues were calculated for up to eight genes located in the vicinity of Gypsy-44 as well as for Gypsy-44 itself in at least two biological replicates. Positions on the metacontig are given. Blue bars indicate fold changes for the non-columnar sample (normalized to 1), red and light pink bars indicate fold changes in the heterozygous and homozygous columnar sample, respectively, for a comparison with the non-columnar sample. Two parallel lines on a bar and the y axis represent a broken axis. Absolute read counts of 0 or 1 in both genotypes of a tissue are considered no expression (n.e.). Error bars in qRT-PCR bar chart represent standard deviations of three technical replicates. The two orange boxes below the two panels of graphs signify zoom-ins on the *dmr6-like* graphs within the region 0 – 2.

In order to identify the link between the columnar-specific Gypsy-44 insertion and the columnar growth habit (most likely corresponding to the first, causal level of differential gene expression) we performed detailed expression analyses of all genes in the vicinity of the Gypsy-44 insertion. Eight genes that had previously been found to be transcribed and were annotated on our BAC metacontig based on the transcriptomic data [10] as well as Gypsy-44 itself were used for in-depth RNA-Seq and qRT-PCR analyses (Table 5, Figure 5). Fold changes were calculated for the primary root datasets as well as for three RNA-Seq Illumina datasets each, generated from shoot apical meristems of A14 and P28 ([13,14], [EMBL: PRJEB2506]) and two transcriptomic datasets each, obtained from the total RNA of a McIntosh and a Wijcik leaf ([10], [EMBL: PRJEB1902]). If all genotypes of one tissue displayed read counts of 0 or 1 for a specific gene, this gene was considered not to be expressed (numeric read counts and fold changes can be found in Additional file 7). The results are shown in the upper part of Figure 5. *At1g08530-like* and *MDP0000934866* (*At1g06150-like*) showed similar expression levels for columnar and non-columnar varieties in all samples analyzed. For *MDP0000927091* (*Autophagy9-like*) and *5NG4-like*, similar levels of transcription were reached in all but the SAM3 dataset, in which they were induced in the columnar sample when compared with the non-columnar sample. Fold changes were less consistent between different tissues and across biological replicates for *MDP0000912172* (*PP2C15-like*), *MDP0000163720* (*ACC1-like*) and Gypsy-44 itself. For the former two genes, this might be caused by the generally lower expression level making fold changes prone to fluctuation. The most striking results were obtained for *dmr6-like* and *MDP0000927098* (*ATL5K-like*), the first two protein coding genes that follow downstream of the Gypsy-44 insertion: they were downregulated in the primary root samples and strongly upregulated in the shoot apical meristem of columnar varieties. These effects were most pronounced for *dmr6-like* in the shoot apical meristem, where no or only basal transcription (0 reads or 1 read) occurred in non-columnar A14, while expression was 30-, 53- and 56-times higher in the three biological replicates of P28.

**Table 5:** **Genes annotated on the BAC metacontig**

**Gene Name**	**Position on BAC Metacontig (strand)**	**Position on Chr 10 of GD Genome**	**Probable Function**
**At1g08530-like**	49164 – 52233 (+)	18743425 – 18746497	unknown
**dmr6-like**	93949 – 96036 (+)	18814292 – 18815452	defense reaction
**MDP0000927098 (ATL5K-like)**	103945 – 104770 (+)	18853768 – 18854596	ubiquitination
**MDP0000927091 (Autophagy9-like)**	119780 – 126200 (−)	18832988 – 18836788	recycling of cell components
**5NG4-like**	140776 – 142998 (−)	18862782 – 18864480	auxin-induced transporter
**MDP0000912172 (PP2C15-like)**	145267 – 146261 (+)	18866768 – 18867762	serine/threonine phosphatase
**MDP0000934866 (At1g06150-like)**	169780 – 175840 (+)	18884175 – 18886818	bHLH transcription factor
**MDP0000163720 (ACC1-like)**	184078 – 185923 (−)	18905698 – 18907541	ethylene biosynthesis

Eight genes were found to be expressed in at least one of the tissues investigated and were annotated on the BAC metacontig. Their name, position on the BAC metacontig and on chromosome 10 of the Golden Delicious (GD) draft genome sequence [36] as well as the possible function (according to BLAST searches) are indicated. bHLH, basic helix-loop-helix.

Gypsy-44 itself, its direct neighboring genes and all other genes within the *Co* target region showing interesting differential gene expression were subjected to qRT-PCRs in order to verify the RNA-Seq results (Figure 5, lower part). Overall, the qRT-PCR results were mostly consistent with the RNA-Seq results. However, fold changes showed less variation across biological replicates in the qRT-PCRs than in the RNA-Seq data. Fold changes of around 1 were obtained for *At1g08530-like* and *MDP0000934866* (*At1g06150-like*) for all tissues with the exception of a 2.5-fold induction of *MDP0000934866* in one of the shoot apical meristem samples. Gypsy-44 was upregulated in the shoot apical meristem, but downregulated in leaves and heterozygous primary roots. *MDP0000163720* (*ACC1-like*) was induced in shoot apical meristems of P28 and slightly induced in heterozygous and homozygous primary roots of one replicate dataset. With regard to the two genes downstream of Gypsy-44, downregulation of *MDP0000927098* (*ATL5K-like*) in columnar primary roots was corroborated, whereas its upregulation in the shoot apical meristem was less pronounced in the qRT-PCR than in the RNA-Seq data. In the qRT-PCR results for *dmr6-like*, downregulation was only detected when heterozygous radicles were compared with non-columnar radicles, but not when homozygous columnar radicles were compared with non-columnar radicles. Strong upregulation was detected in the Wijcik leaf when compared with the McIntosh leaf. The very strong induction of *dmr6-like* in the shoot apical meristem samples was validated by a 41-fold and 202-fold induction in the columnar shoot apical meristem replicates.

**Figure 6:**
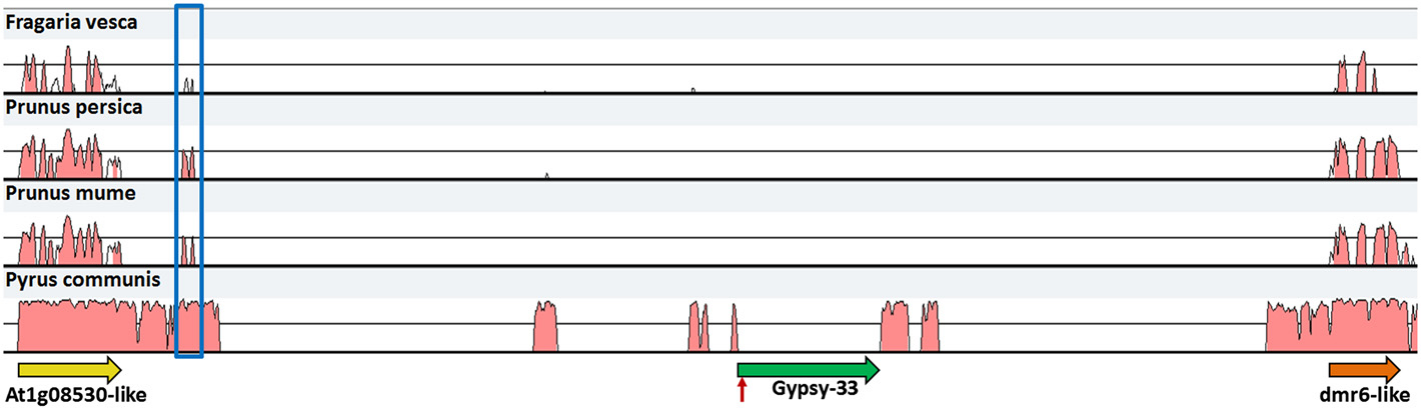
**Conserved regions within the Gypsy-44 region.** An mVISTA plot of sequence identity between the *Malus* x *domestica* sequence spanning the region from *At1g08530-like* to *dmr6-like* (x axis) and different Rosaceae species indicates sequence conservation in exon regions and one CNS present in all species investigated (blue box). Other conserved regions in Pyrus correspond to TEs that probably inserted before the divergence of pear and apple. The red arrow marks the Gypsy-44 insertion site.

In order to identify any conserved cis-regulatory sequences of *dmr6-like* whose effect might be impaired by the insertion of Gypsy-44, we conducted comparative sequence analyses among the Rosaceae species apple, pear (*Pyrus communis*), peach (*Prunus persica*), Chinese plum (*Prunus mume*) and strawberry (*Fragaria vesca*). In these species, the genes flanking the Gypsy-44 insertion are microcolinear (with an inverse orientation on linkage group 2 of *Fragaria*), enabling the analysis of conserved non-coding sequences (CNSs) in the intergenic region. Remarkably, the intergenic region between *At1g08530-like* and *dmr6-like* or their orthologs has a size of about 33 kb in *Malus* (without the additional 8.2 kb of Gypsy-44), 6.8 kb in *Pyrus*, 1.7 kb in *Fragaria* and 1.4 kb in *Prunus*, suggesting that it has served as a popular target of TE insertions in the Pyreae and especially in the *Malus* lineage. Within this region, one CNS of about 400 bp showing two peaks in the identity plot could be observed about 31 kb upstream of *dmr6-like* in *Malus* (Figure 6). The sequence of the second peak region yielded a hit against a class II TE of the Mariner group in a CENSOR BLAST search [40], whereas no information could be obtained from database searches for the first part, suggesting that it might contain sequences conserved owing to their importance for gene regulation.

In conclusion, there is evidence that Gypsy-44 influences the expression level of its direct downstream gene, *dmr6-like*, and most likely also some genes located further downstream, possibly via impairment of the function of a CNS.

## Discussion

### Genotyping, sequencing and assemblies

So far, only heterozygous columnar cultivars have been described in the available literature, with one exception of a homozygous cultivar at Geisenheim University [10]. Therefore, Meulenbroek et al. [4] suggested that *Co* or a linked gene might negatively influence the fitness of pollen, seeds or early seedlings. However, crosses between two columnar apple trees have been shown to yield up to 75% columnar progeny [4,41], which is in accordance with the result of dominant Mendelian inheritance comprising 25% homozygous and 50% heterozygous columnar plants. In our genotyping experiments with the progeny of a heterozygous columnar cultivar we detected 17% homozygous columnar radicles. Hence, homozygous columnar primary roots are clearly viable. They did not show any deviating phenotypic differences either. The genotype ratio of non-columnar : heterozygous columnar : homozygous columnar of 2 : 3 : 1 is not really surprising because seeds were obtained by open pollination.

In this study, we detected that apple primary roots express between 9,000 and 32,000 distinct genes (represented as contigs yielding different BLAST hits). The number of genes identified as expressed is dependent on the dataset and the criteria used to distinguish individual genes. Assembling the smaller replicate datasets produced less contigs than assembling the larger datasets because increased sequencing depth facilitates detection of a higher number of transcripts. Regarding the total number of active genes in primary roots, we consider the numbers obtained from BLAST searches against the Malus Unigene dataset, around 17,000, to be the best estimate for those genes that are assembled. MDP and EST most likely show redundancy, listing alleles or fragments of one gene as individual genes, and the SwissProt/UniProtKB probably does not contain all the apple-specific genes. However, considering that not all contigs matched to a Malus Unigene entry and that not all reads were assembled into contigs, the actual number of active genes is probably significantly higher than estimated. There is still a lot of discussion about the real number of genes in apple. Within its genomic sequence, almost 58,000 genes have been anchored, which is the highest gene number reported for plants so far, and this has even been considered an underestimate [36]. However, pear only has about 42,000 genes, and when the apple genome is re-assembled filtering out overlapping genes in apple chromosomes that might correspond to alleles instead of individual genes, the gene number drops down to 45,293 [42]. This would be more consistent with gene numbers of other close relatives such as peach (27,852) [43] and strawberry (34,809) [44]. Newcomb et al. [45] conducted one of the first exhaustive EST analyses in apple and found about 43,000 non-redundant sequences, which they considered to be approximately half of the apple genes. By contrast, Allan et al. [46] assumed the apple EST dataset of 68,599 sequences from databases to be an overestimate. On the other hand, based on EST analyses by Sanzol [47], 68% of the apple genes fall into families with a mean copy number of 4.6 owing to several genome duplications, and the members of one family can have high sequence similarity but still represent different genes rather than alleles. Hence, the exact gene number remains debatable.

With regard to gene function, the well-known non-model-organism problem of an unsatisfying proportion of genes being successfully annotated is encountered [48]. We were able to assign GO terms to about 35% of contigs. This is slightly lower than the percentage of contigs annotated in RNA-seq studies of most other non-model organisms such as 50% in ten different invertebrates [49], 47 and 48% in the non-model plants *Streptocarpus rexii* and olive, respectively [50,51], and 40% in the bloom-forming alga *Emiliania huxleyi* [52]. However, it is much higher than the 15 – 19% of contigs contigs that could be successfully annotated in the non-model gastropod *Nerita melanotragus* [53]. Further efforts would most likely enable the assignment of possible functions to a higher number of contigs, similar to the techniques applied by [50]. Of those genes that were annotated, the majority had the expected function in growth, development and signaling, which is in agreement with the results obtained from transcriptome analyses of the poplar root and the *Arabidopsis* pericycle, where the most highly expressed genes were found to be involved in protein synthesis, metabolism, cellular communication and signal transduction [20,22].

### Evaluation of differential gene expression

DESeq was used to evaluate differential gene expression since it has been found to perform well in a comparison of DESeq, edgeR, baySeq and a method employing a two-stage Poisson model [54]. The comparison of gene expression in columnar and non-columnar radicles yields a lower number of significantly differentially expressed genes than the comparison of gene expression in columnar and non-columnar shoot apical meristems [13]. Differential expression was detected for 200 – 300 and more than 600 genes in primary roots and shoot apical meristems, respectively, despite a more stringent definition of significant differential expression in the shoot apical meristem study of Krost et al. [13]. This might indicate that gene expression of columnar and non-columnar varieties is more similar in underground than in aerial organs. Alternatively, the difference in gene activity might be smaller in early developmental stages than at a higher age of the plants, which would explain the fact that the columnar growth habit can only be reliably detected after about 2 – 3 years [55,56]. However, interpretation of the differential expression data is hampered by the fact that the genetic background of the individual radicles used for RNA extraction is unclear due to the open pollination of the flowers. While the mother plant is P28, the father could be any of the dozens of different varieties grown on the field. Additionally we have no knowledge about recombination that might have occurred within the gametes. We chose this material because we wanted to find out whether homozygous columnar individuals occur under natural field conditions. However, for gene expression analyses seeds obtained from targeted crossings would be much more favorable. Moreover, radicles consist of distinct developmental regions in the longitudinal direction and radicles were halved without detailed prior investigation so that different developmental zones most likely harboring different gene expression patterns might have been grouped to each RNA isolation sample. In future studies, the radicles should be subdivided into their individual zones by microscopic control (possibly after fluorescence *in situ* hybridization against marker genes of the distinct zones, similarly to [17]) and the gene expression should be investigated separately for each zone. Nevertheless, due to the high degree of correlation across samples with regard to active genes (see Additional file 2) and read counts for each gene (Table 4), we are confident that we analyzed samples of similar developmental zones and stages.

The pattern of differential gene expression in primary roots is in concordance with that of aerial plant parts. The differential expression of genes involved in cell wall biosynthesis and modification observed for heterozygous and homozygous columnar primary roots has already been described for shoot apical meristems of columnar trees [14]. In addition, an increased transcription of genes associated with jasmonate signaling and biosynthesis has been found for shoot apical meristems and leaves of columnar varieties [10,13,14], and elevated ethylene signaling and biosynthesis seem to occur not only in columnar radicles, but also in leaves of columnar Wijcik compared with non-columnar McIntosh [10]. Therefore, alterations in cell boundary components and an increased level of jasmonates and ethylene might represent features of columnar apple trees that are already established in the early developmental stages and across different organs of the plantlet.

### The first level of differential gene expression occurs in the Gypsy-44 vicinity

In a previous study [10] we found that columnar growth is most likely caused by the insertion of the Gypsy-44 LTR retrotransposon on chromosome 10 at 18.8 Mb, in accordance with the target region determined for the *Co* gene by Moriya et al. [57] and Baldi et al. [56]. Since it is a nested retrotransposon insertion, the link between its presence and the formation of the columnar phenotype remains to be unraveled. In this study, gene expression of Gypsy-44 itself and its neighboring genes was found to differ in columnar compared with non-columnar varieties in the individual plant organs. The expression of Gypsy-44 is induced in the shoot apical meristem. Its nearest downstream neighbor gene, *dmr6-like*, shows very strong (up to 200-fold) upregulation in shoot apical meristems and downregulation in the primary root samples of columnar individuals. The next gene downstream of *dmr6-like*, *MDP0000927098* (*ATL5K-like*), and one other downstream gene, *MDP0000163720* (*ACC1-like*), are also induced in shoot apical meristems of columnar P28. In contrast, expression of the upstream neighbor of Gypsy-44, *At1g08530-like*, remains unchanged. This indicates that Gypsy-44 influences the gene expression pattern of the downstream genes, especially *dmr6-like*, which has already been discussed as a possible candidate gene for the mediation of the columnar phenotype [10,12]. In addition to its induction in axillary meristems [12] and shoot apical meristems [10], *dmr6-like* is induced in our Wijcik leaf qRT-PCR samples, which were generated from RNA of the newly developing leaves near the top of the tree, but it shows no expression in the Wijcik leaf RNA-Seq data, which were obtained from RNA of young but fully developed leaves. We therefore conclude that *dmr6-like* is upregulated in all aerial organs of columnar trees that are important for the development of the plant organs showing differences between columnar and non-columnar plants: the shoot apical meristem, which is responsible for the formation of the short internodes and short lateral branches, the axillary meristem, which does not develop into lateral branches but instead produces short spurs, and the young leaves, which form a thicker palisade parenchyma with a high chlorophyll content [58]. In contrast, this upregulation does not occur in organs that have finished their development, such as older leaves, as well as in primary roots, which are less significant for the growth habit of the aerial organs; instead, a reverse regulation seems to take place on the below-ground organs. Wolters et al. [12] have already overexpressed *dmr6-like* in *Arabidopsis* and found the plants to have shorter internodes and smaller branches, strongly suggesting that *dmr6-like* is involved in the formation of the columnar growth habit. Our results support this hypothesis, but also raise further questions, e.g. how the tissue specificity of this induction is achieved.

Possible effects of the Gypsy-44 insertion like alterations in the methylation pattern or the allocation of enhancers and/or silencers have already been discussed [10]. The tissue specificity of this effect suggests that the Gypsy-44 insertion with a size of 8.2 kb might move a tissue-specific silencer of *dmr6-like* that is normally active in apical and axillary meristems and young developing leaves to a distance from the gene that abolishes silencer function, resulting in upregulation of *dmr6-like*. Alternatively, an enhancer being active in those tissues crucial for development might be moved into spatial proximity of *dmr6-like* due to an altered chromosome architecture (e.g. looping of the Gypsy-44 containing region) and thereby cause induction in tissues where *dmr6-like* is normally not expressed. We identified a candidate for a CNS that might have a role in gene regulation at 31.4 kb distance upstream of *dmr6-like*. This is reminiscent of the situation for the *vgt1* locus in maize, a CNS positioned 70 kb downstream of *ZmRap2.7. ZmRap2.7* is a repressor of flowering and its expression is downregulated in varieties that carry a miniature inverted repeat TE (MITE) insertion within *vgt1*, resulting in early flowering [59]. However, in our case the TE insertion does not occur at the CNS site, but downstream of it. This might still influence its function due to the increased distance between the CNS and *dmr6-like*. The fact that the region crucial for the formation of columnar growth, including *dmr6-like*, is conserved across several Rosaceae species might provide an opportunity for the creation not only of new columnar apple cultivars, but also of columnar pear and plum varieties by genetic engineering in future.

In *Arabidopsis*, *dmr6* loss-of-function mutants show enhanced expression of a subset of defense-associated genes, suggesting a control different from that in apple [60]. Interestingly, the truncated protein of the *dmr6-1* mutant is constitutively expressed in the shoot apical meristem and leaf primordia of *Arabidopsis* [60], in line with the expression profile of *dmr6-like* in columnar apple trees. The regulatory pathways influenced by columnar growth downstream of the *dmr6-like* upregulation still await elucidation. Since *dmr6-like* is associated with downy mildew resistance in *Arabidopsis* [60] it might trigger a defense response leading to a diseased-looking growth habit. On the other hand, members of the 2OG Fe(II) oxygenase family are involved in the biosynthesis of ethylene, gibberellins and flavonoids, the latter of which modulate polar auxin transport, so that upregulation of *dmr6-like* might directly influence phytohormone concentrations [12,61-64]. In apple, silencing of the chalcone synthase, the first committed enzyme in flavonoid biosynthesis, leads to decreased concentrations of flavonoids and causes altered cellular organization and cell wall composition, increased auxin transport and a reduction of plant size due to shortened internodes [65]. Specifically some genes encoding indole-3-acetic acid (IAA) transporters show upregulation in shoot apical meristem of P28 compared with A14, in line with an altered auxin transport capacity [13].

Furthermore, besides the induction of *dmr6-like*, other gene expression changes in the Gypsy-44 region or the upregulation of Gypsy-44 itself in shoot apical meristems might play a role in phenotype generation. Induction of *MDP0000163720* in columnar shoot apical meristems, which encodes a homolog of Aminocyclopropane-1-carboxylate oxidase, might lead to increased levels of ethylene. Since ethylene crosstalks with many other phytohormones such as auxin, cytokinin, abscisic acid, jasmonates and salicylic acid (for example [66-70]), this could contribute to the altered hormonal balance in columnar trees.

## Conclusions

Open pollination of heterozygous columnar apple trees yield non-columnar, heterozygous columnar and homozygous columnar seedlings in a genotype ratio of 3 : 2 : 1. This suggests that homozygous columnar plants are viable within their early life stages and the lack of described homozygous columnar varieties is probably due to breeders’ selection criteria. Apple primary roots display a transcriptome profile typical for a developing and actively growing tissue. About 200 – 300 genes are differentially regulated in columnar compared with non-columnar radicles, and these are involved in cell wall synthesis and modification, stress reactions and phytohormone signaling and biosynthesis. The molecular cause of columnar growth, the Gypsy-44 insertion, leads to upregulation of its nearest downstream gene, *dmr6-like*, in all tissues that are crucial to the development of the visible plant growth habit. The role of *dmr6-like* in pathogen resistance possibly results in upregulation of defense reactions in aerial plant organs leading to a phenotype reminiscent of a diseased plant. Alternatively or additionally, the upregulation of *dmr6-like* might lead to alterations in flavonoid content causing altered auxin transport, which would be in line with changes in auxin signaling and metabolism that have been detected in previous studies. The Gypsy-44 insertion is the causative event, then leading to upregulation of *dmr6-like* as a second step. In columnar primary roots, however, *dmr6-like* is downregulated, so that the activity of a tissue-specific transcriptional regulator might play a pivotal role. We are confident that our data will contribute to the elucidation of the whole signaling cascade causing columnar growth in apples.

## Methods

### Plant material

For primary root transcriptome studies, apples from the heterozygous columnar cultivar P28 obtained by open pollination on a field with many columnar apple trees were collected in August 2011 at Geisenheim University. Seeds were extracted and dried at room temperature for about 1 – 2 weeks.

For qRT-PCRs, shoot tips of columnar P28 and non-columnar ‘A14-190-93K’ (A14) sampled on September 29, 2009 and very young apical leaves of ‘McIntosh’ and ‘McIntosh Wijcik’ sampled on July 19, 2013 were snap frozen in dry ice or liquid nitrogen, respectively, and stored at −80°C until further use.

### Seed germination

Dried seeds were swabbed with 70% ethanol to roughly remove contamination and were placed in Petri dishes equipped with towel-paper that had been moistened with tap water. The petri dishes were wrapped in tinfoil and were stored in the dark at 4°C for about 12 weeks. When the radicles reached a length of about 2 – 3 cm they were snap frozen in liquid nitrogen, cut off the seed, cut in half and stored at −80°C until further use.

### DNA isolation

The upper half of each radicle was subjected to DNA extraction according to a manual protocol. One radicle was transferred into 500 μL Cetyltrimethyl ammonium bromide (CTAB) extraction buffer, to which 168 μL 8 M urea and a spatula tip of polyvinylpyrrolidone (PVPP) were added. The radicle was thoroughly homogenized with a micropestle followed by incubation at 65°C for 30 min. After centrifugation (10 min at 13,000 rpm and room temperature) the supernatant was purified by three chloroform isoamyl alcohol (24:1) extractions and subsequent ethanol precipitation.

### Primary root genotyping

In order to investigate the genotype of each radicle with regard to the presence of the Gypsy-44 retrotransposon associated with the columnar growth habit, PCRs were performed with primers spanning the left border, the right border or the whole TE insertion as described by Otto et al. [10]. Results were double checked by PCRs with our indel-based markers I2_3_M1 and H1_M1 [37]. All primers were purchased from Invitrogen^TM^ (Invitrogen, Darmstadt, Germany). PCRs were carried out in 50 μL reaction volume containing at least 10 ng of radicle DNA and 1 U Go-Taq polymerase (Promega, Madison, United States) as follows: initial denaturation at 94°C for 5 min followed by 40 amplification cycles of denaturation at 94°C for 30 sec, annealing at 58.4°C or 60°C for 30 sec and elongation at 72°C for 30 sec. A final elongation step at 72°C for 10 min was included. PCR products were analyzed on horizontal 2% agarose gels.

### RNA extraction

The distal halves (tips) of radicles were used for RNA extraction. For the initial Illumina RNA-Seq, one radicle of each genotype was subjected to total RNA isolation. For replicate datasets, three frozen radicles of one genotype were pooled prior to RNA extraction. Total RNA was isolated using the innuPREP Plant RNA Kit (Analytik Jena, Jena, Germany) following the manufacturer’s instructions. A DNase I (Fermentas, St. Leon-Roth, Germany) digestion step on the column was included to remove genomic DNA. RNA concentration and integrity were assessed with Bioanalyzer RNA Nano or Pico chips (Agilent, Santa Clara, USA).

For qRT-PCRs with leaf and shoot tip material, samples were transferred to liquid nitrogen, disrupted with a mortar and pestle and RNA was isolated using the innuPREP Plant RNA kit (Analytik Jena, Jena, Germany) following the manufacturer’s instructions.

### Illumina sequencing

For RNA-Seq library construction 500 ng of total RNA of each primary root sample were sent to GENterprise Genomics (Mainz, Germany). RNA Integrity Number (RIN) values were 7.6, 7.8 and 8.3 for the total RNA obtained from the single non-columnar, heterozygous columnar and homozygous columnar radicle, repectively. The RNA of the replicate samples had RIN values of 8.2, 7.0 and 7.0 for the non-columnar, the heterozygous columnar and the homozygous columnar genotype, respectively. 100 bp paired-end runs were conducted on the Illumina HiSeq 2000 or Illumina HiSeq 2500 (Illumina, San Diego, USA) of the IMSB Mainz.

### Assembly of Illumina data

Prior to assembly, Illumina raw data were trimmed with an in-house Perl script to remove low quality stretches. 6 bp at the 5’ end and 5 bp at the 3’ end were cut off, and bases with a Phred score < 20 or called as “N” were removed. After trimming, only sequences with a minimum length of 30 bp were maintained. All remaining sequences were assembled with the CLC Assembly Cell (CLC bio, Aarhus, Denmark) using different k-mer sizes and a bubble size of 300. The assemblies yielding the highest N50 value were used for BLAST searches. To evaluate these assemblies, trimmed reads were mapped back to contigs with Bowtie [71], allowing no mismatches.

### BLAST searches

Contigs generated by assembling were subjected to BLAST searches [33] against 1) MDPs representing the genes annotated in the Golden Delicious genome sequence [36] as extracted from the Genome Database of Rosaceae (http://www.rosaceae.org/), 2) the NCBI Malus expressed sequence tag (EST) dataset, 3) the Malus Unigene v5.0 dataset representing filtered ESTs assembled with CAP3 [72], available at the Genome Database of Rosaceae (http://www.rosaceae.org/), and 4) the NCBI SwissProt/UniProtKB database, all databases downloaded on February 3, 2014. BLAST results were filtered and analyzed at an E-Value cutoff of 10^−5^.

### Blast2GO

Results of BLAST searches with the replicate datasets against SwissProt/UniProtKB were imported into CLC Genomics Workbench v6.5 (CLC bio, Aaarhus, Denmark) and analyzed with the Blast2GO plugin using default parameters [34,35]. Following GO Slim reduction, combined graphs of the GO term biological process were created and were converted to pie charts of the second-level terms.

### RNA-Seq analysis

Illumina raw sequencing data were imported into CLC Genomics Workbench v6.5 (CLC bio, Aaarhus, Denmark) as paired-end reads and were subjected to trimming and RNA-Seq mappings against our BAC metacontig of the transposon region or the Golden Delicious reference genome as previously described [10]. A similarity of at least 98% was demanded for at least 90% of the read length and using the “include broken pairs” counting scheme. For visualization of differential gene expression between two datasets each, total gene read counts for each MDP were extracted from RNA-Seq mapping results and analyzed in DESeq [38] with default parameters and including the biological replicates. Only genes with base mean > 100, fold change > 2 (or < 0.5) and p-value < 0.05 were maintained, and genes were ranked according to their p-values. Subsequently, the LOG2 fold changes were loaded into MapMan [39] and mapped against the *Malus* x *domestica* mapping file.

### qRT-PCRs

Genes showing interesting differential regulation were subjected to qRT-PCR analysis in order to verify the bioinformatics results. Primers were designed to span the last intron of each gene and were purchased from Invitrogen (Darmstadt, Germany). A list of primers can be found in Additional file 8. 800 ng of total RNA extracted from three pooled primary roots of each genotype (RIN > 7) and 200 ng of human MDA-MB468 RNA serving as exogenous control were converted to cDNA using SuperScript III Reverse Transcriptase (Invitrogen, Darmstadt, Germany) and 100 μM oligo(dT)_18_VN primer according to the manufacturer’s instructions. In order to compare results of different tissues, the same procedure was applied to RNA extracted from shoot tips (shoot apical meristems) of A14 and P28 and to RNA isolated from young leaves of McIntosh and McIntosh Wijcik. All qRT-PCR experiments were carried out in a 7500 Fast Real-Time PCR System using Power SYBR Green PCR Master Mix (Applied Biosystems, Carlsbad, USA). Reactions were conducted in triplicate and two biological replicates were analyzed.

### Conserved sequence analysis

Orthologs were retrieved from pear, peach, Chinese plum (Mei) and strawberry based on BLASTx searches yielding PCP006734.1 and PCP039177.1 (pear), ppa009607m and ppa014841m (peach), Pm020608 and Pm020609 (Chinese plum) and mrna08066.1-v1.0-hybrid and mrna08065.1-v1.0-hybrid (strawberry) as the best hits for At1g08530-like and dmr6-like, respectively. The corresponding genomic sequences were extracted from GDR and were aligned in mVISTA (http://genome.lbl.gov/vista/index.html) [73] with parameters “calculated window” of 100 bp, “minimum consensus width” 100 bp and “consensus identity” 70%.

### Availability of supporting data

Transcriptomic Illumina data of primary roots, SAMs and leaves can be found in the EMBL EBI Short Read Archive under study accession numbers PRJEB6212 (http://www.ebi.ac.uk/ena/data/view/PRJEB6212), PRJEB2506 (http://www.ebi.ac.uk/ena/data/view/PRJEB2506) and PRJEB1902 (http://www.ebi.ac.uk/ena/data/view/PRJEB1902), respectively. The annotated genomic contig of the Gypsy-44 region including all annotated genes can be retrieved from the EMBL EBI GenBank repository under accession number HF968765 (http://www.ebi.ac.uk/ena/data/view/HF968765). All other supporting data can be found in the supplementary online material of this article.

## Additional files

Additional file 1:
**Pie charts of level 2 GO terms assigned to contigs of primary root transcriptome assemblies.** Assemblies of primary root transcriptome Illumina reads were subjected to BLAST searches against SwissProt/UniProtKB and subsequent Blast2GO analysis. Pie charts of level 2 GO terms are shown for data obtained from three pooled non-columnar **(A)**, heterozygous columnar **(B)** and homozygous columnar **(C)** primary roots. Additional file 2:
**Venn diagram of active genes in the datasets of the non-colmnar, heterozygous and homozygous columnar primary roots.** Annotated MDPs to which at least one read of the original or replicate RNA-Seq dataset of primary roots could be mapped were considered active. Active genes were compared across genotypes. 46,417 genes are active in all three genotypes, 1385 genes are active in the non-columnar and the heterozygous columnar samples, but not in the homozygous colmnar samples. 1005 genes are expressed in the non-clumnar and the homozygous columnar roots, but not in the heterozygous columnar roots. 655 active genes are shared between heterozygous and homozygous columnar primary roots and are not expressed in non-columnar radicles. 630, 430 and 2845 genes are expressed only in the non-columnar, the hetrozygous and the homozygous columnar radicles, respectively. Additional file 3:
**Table of significantly differentially expressed genes in heterozygous compared with non-columnar primary roots.** Total gene reads of the two biological replicate data were imported into DESeq and analysed for differential expression. Only genes with base mean > 100, fold change > 2 and p-value < 0.05 are displayed. The number of the MapMan BIN each gene was assigned to as well as the gene name, identifier (PAC-ID) and description are given as in the MapMan mapping file for apple. Additional file 4:
**Table of significantly differentially expressed genes in homozygous columnar compared with non-columnar primary roots.** Total gene reads of the two biological replicate data were imported into DESeq and analyzed for differential expression. Only genes with base mean > 100, fold change > 2 and p-value < 0.05 are displayed. The number of the MapMan BIN each gene was assigned to as well as the gene name, identifier (PAC-ID) and description are given as in the MapMan mapping file for apple. Additional file 5:
**Figure of differentially expressed genes in homozygous columnar compared with heterozygous primary roots.** LOG2 fold changes of significantly differentially expressed genes (normalized to the heterozygous sample) as listed in Additional File 6 were imported and were visualized in MapMan for the homozygous columnar sample with regard to a metabolism overview (A), pathogen/pest attack (B) and transcription and translation (C). Genes upregulated in homozygous columnar radicles are shown as red boxes, downregulated genes are shown as blue boxes. Additional file 6:
**Table of significantly differentially expressed genes in homozygous columnar compared with heterozygous primary roots.** Total gene reads of the two biological replicate data were imported into DESeq and analyzed for differential expression. Only genes with base mean > 100, fold change > 2 and p-value < 0.05 are displayed. The number of the MapMan BIN each gene was assigned to as well as the gene name, identifier (PAC-ID) and description are given as in the MapMan mapping file for apple. Additional file 7:
**Table of total read counts and fold changes of genes and Gypsy-44 within the** ***Co*** **target region.** Total Read counts of RNA-Seq data are displayed. Additional file 8:
**Primer List.** Primers used for marker PCRs and qRT-PCR.

## Abbreviations

ACC1: 1-aminocyclopropane-1-carboxylate synthase 1
BLAST: Basic Local Alignment Search Tool
*Co*: *Columnar*
CNS: conserved non-coding sequence
CTAB: cetyltrimethyl ammonium bromide
*dmr6*: *Downy mildew resistant 6*
EST: Expressed sequence tag
GO: Gene Ontology
IAA: Indole-3 acetic acid
LTR: Long terminal repeat
MDP: *Malus* x *domestica* protein
PP2C: Protein phosphatase 2C
PVPP: Polyvinylpyrrolidone
qRT-PCR: Quantitative real-time polymerase chain reaction
