# Supplementary figures and images for: Columnar apple primary roots share some features of the columnar-specific gene expression profile of aerial plant parts as evidenced by RNA-Seq analysis
Source: BMC Plant Biol. 2015 Feb 4;15:34. doi: 10.1186/s12870-014-0356-6 (PMC4352258; doi:10.1186/s12870-014-0356-6)

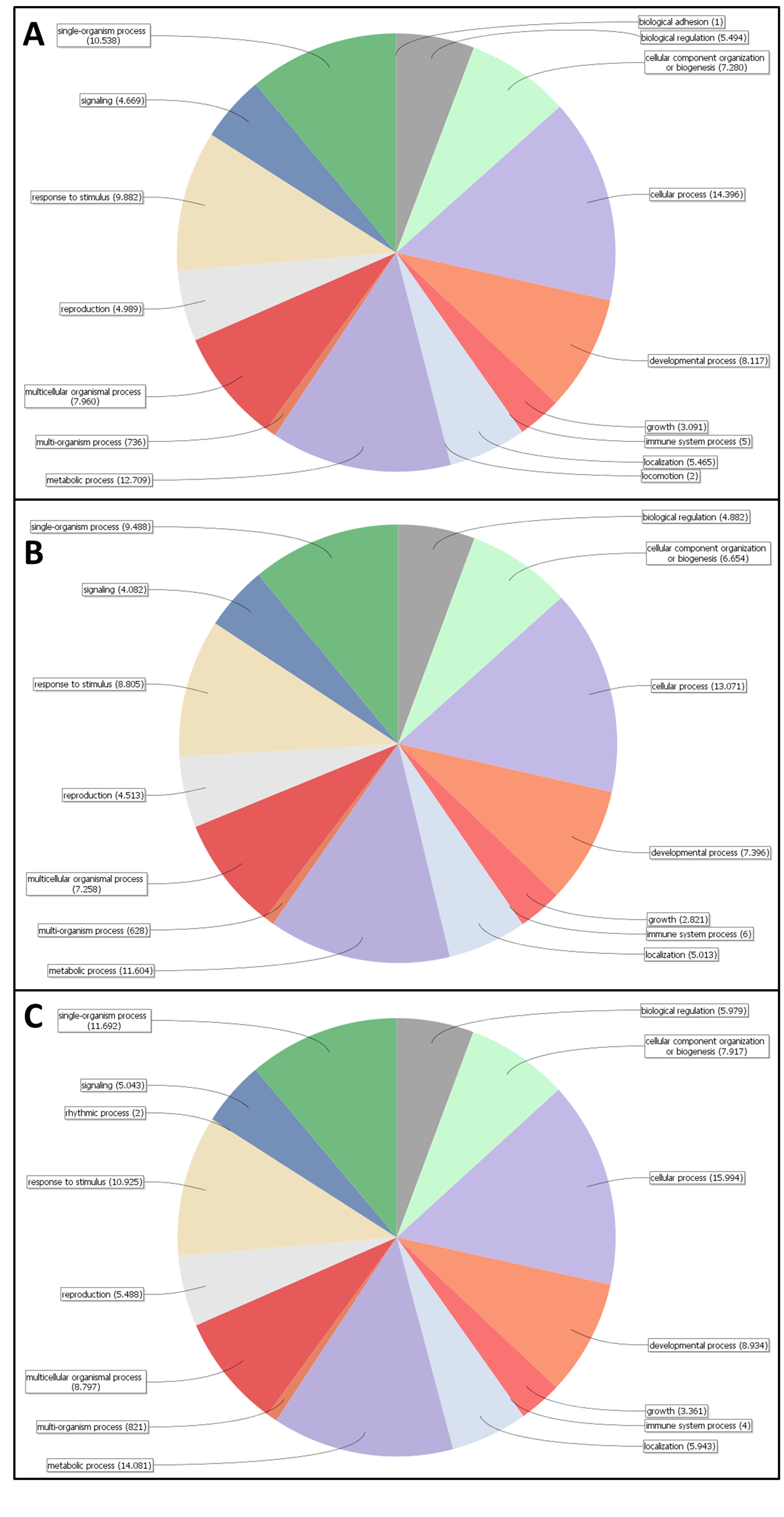

Supplement: Additional file 1: — Pie charts of level 2 GO terms assigned to contigs of primary root transcriptome assemblies. Assemblies of primary root transcriptome Illumina reads were subjected to BLAST searches against SwissProt/UniProtKB and subsequent Blast2GO analysis. Pie charts of level 2 GO terms are shown for data obtained from three pooled non-columnar (A), heterozygous columnar (B) and homozygous columnar (C) primary roots. [file 12870_2014_356_MOESM1_ESM.png]

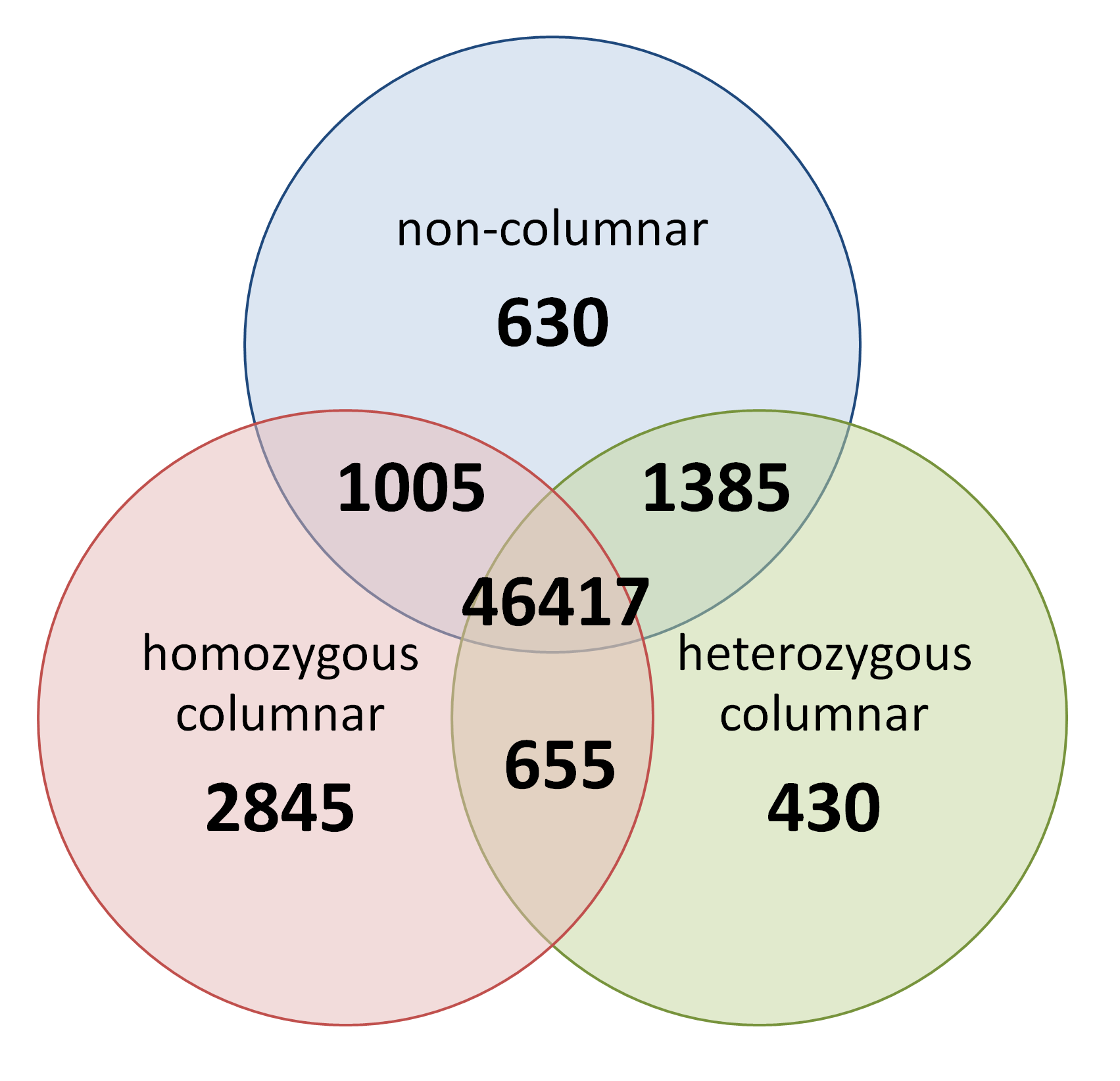

Supplement: Additional file 2: — Venn diagram of active genes in the datasets of the non-colmnar, heterozygous and homozygous columnar primary roots. Annotated MDPs to which at least one read of the original or replicate RNA-Seq dataset of primary roots could be mapped were considered active. Active genes were compared across genotypes. 46,417 genes are active in all three genotypes, 1385 genes are active in the non-columnar and the heterozygous columnar samples, but not in the homozygous colmnar samples. 1005 genes are expressed in the non-clumnar and the homozygous columnar roots, but not in the heterozygous columnar roots. 655 active genes are shared between heterozygous and homozygous columnar primary roots and are not expressed in non-columnar radicles. 630, 430 and 2845 genes are expressed only in the non-columnar, the hetrozygous and the homozygous columnar radicles, respectively. [file 12870_2014_356_MOESM2_ESM.png]

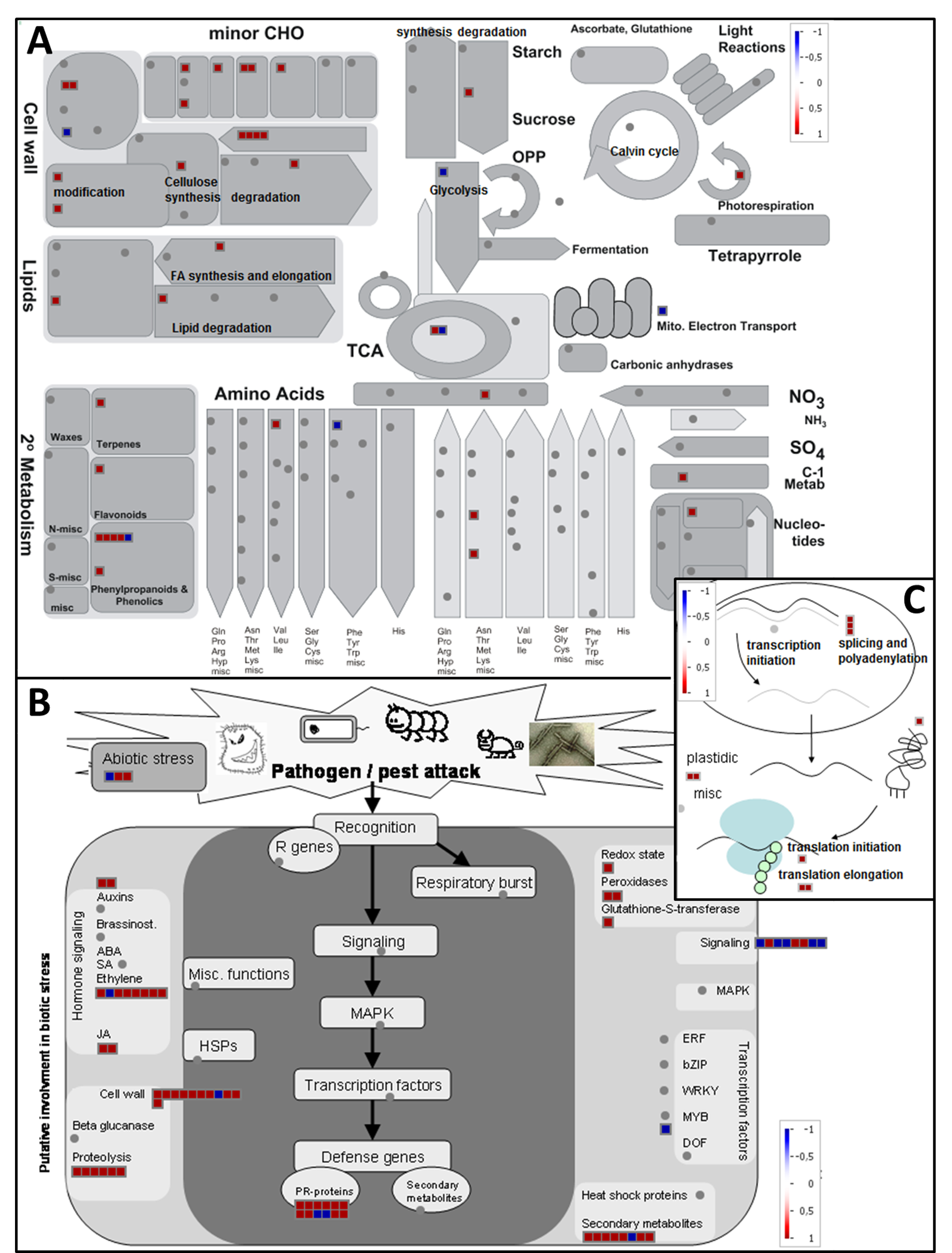

Supplement: Additional file 5: — Figure of differentially expressed genes in homozygous columnar compared with heterozygous primary roots. LOG2 fold changes of significantly differentially expressed genes (normalized to the heterozygous sample) as listed in Additional File 6 were imported and were visualized in MapMan for the homozygous columnar sample with regard to a metabolism overview (A), pathogen/pest attack (B) and transcription and translation (C). Genes upregulated in homozygous columnar radicles are shown as red boxes, downregulated genes are shown as blue boxes. [file 12870_2014_356_MOESM5_ESM.png]
